# RIP-Seq of EZH2 Identifies **TCONS-00036665** as a Regulator of Myogenesis in Pigs

**DOI:** 10.3389/fcell.2020.618617

**Published:** 2021-01-12

**Authors:** Shanshan Wang, Xuewen Xu, Yan Liu, Jianjun Jin, Feng Zhu, Wei Bai, Yubo Guo, Jiali Zhang, Hao Zuo, Zaiyan Xu, Bo Zuo

**Affiliations:** ^1^Key Laboratory of Swine Genetics and Breeding of the Ministry of Agriculture and Rural Affairs, Huazhong Agricultural University, Wuhan, China; ^2^Key Laboratory of Agriculture Animal Genetics, Breeding and Reproduction of the Ministry of Education, Huazhong Agricultural University, Wuhan, China; ^3^College of Animal Science, South China Agricultural University, Guangzhou, China; ^4^The Cooperative Innovation Center for Sustainable Pig Production, Wuhan, China

**Keywords:** EZH2, lncRNA, pig, myogenesis, epigenetics

## Abstract

Enhancer of zeste homolog 2 (EZH2) is the catalytic subunit of polycomb repressive complex 2 and contains a SET domain that catalyzes histone H3 trimethylation on lysine 27 (H3K27me3) to generate an epigenetic silencing mark. EZH2 interacts with transcription factors or RNA transcripts to perform its function. In this study, we applied RNA immunoprecipitation sequencing and long intergenic non-coding RNA (lincRNA) sequencing methods to identify EZH2-binding lincRNAs. A total of 356 novel EZH2-binding lincRNAs were identified by bioinformatics analysis and an EZH2-binding lincRNA *TCONS-00036665* was characterized. *TCONS-00036665* promoted pig skeletal satellite cell proliferation but inhibited cell differentiation, and this function was conserved between pigs and mice. Further mechanistic studies indicated that *TCONS-00036665* can bind to EZH2 and recruits EZH2 to the promoters of the target genes *p21*, *MyoG*, and *Myh4*, which leads to the enrichment of H3K27me3 and the repression of target gene expression and pig myogenesis. In conclusion, the lincRNA *TCONS-00036665* regulates pig myogenesis through its interaction with EZH2.

## Introduction

Vertebrate skeletal muscle myogenesis is a complex process, which requires a variety of factors to work together. During embryonic development, most skeletal muscle cells originate from dermomyotome. They are termed myogenic progenitor cells and are characterized by high expression of the paired-box transcription factors Pax3 and Pax7 (Hindi et al., 2013), and migrate to the limbs and trunk. Pax3 is necessary for the formation of limb muscles, affecting either the generation of myogenic progenitor cells in the somitic dermomyotome or the migration of these cells into the limb field (Bober et al., 1994). Pax3 and Pax7 can induce expression of myogenic regulatory factors (MRFs) Myf5 and MyoD, thus promoting the specification of muscle progenitor cells to committed myoblasts (Maroto et al., 1997; Buckingham and Relaix, 2007; Lagha et al., 2008). As muscle progenitor cells enter myogenesis, Pax3 and Pax7 are down-regulated, and Myf5, Mrf4, and MyoD are activated; subsequent muscle differentiation depends on MyoD, Mrf4, or MyoG (Buckingham, 2006). In the mouse, Myf5 gene is the first MRF to be expressed in the somite with a role in the early events of myogenic differentiation (Ott et al., 1991). MyoD is also required for the determination of skeletal myoblasts (Rudnicki et al., 1993). Myf5/MyoD double mutant mice are devoid of muscle fiber and myoblasts, while MyoD null or Myf5 null mice develop normal skeletal muscle (Rudnicki et al., 1992; Braun and Arnold, 1996), indicating that Myf5 and MyoD can compensate for each other. MyoG and Myf6 were expressed in a genetic pathway downstream of Myf5 and MyoD. MyoG null mice have virtually no muscle fibers but myoblasts appear normal (Hasty et al., 1993; Nabeshima et al., 1993), suggesting that MyoG is necessary for terminal differentiation. Upon postnatal muscle development, a population of mononucleated precursors, called satellite cells, can generate new myonuclei (Beauchamp et al., 2000) and promote myofiber growth. In mature muscle, satellite cells are mitotically quiescent (Schultz et al., 1978), quiescent satellite cells express Pax7 and Myf5, but not MyoD or MyoG (Yin et al., 2013). Once the muscle is injured, satellite cells can be rapidly activated and give birth to myogenic precursor cells; these cells express Myf5 and MyoD, and later express MyoG for a series of proliferation, differentiation, and fusion to form new myofibers (Kuang et al., 2007; Yin et al., 2013); a part of activated satellite cells also produce offspring that restore quiescent satellite cell pool.

Polycomb group (PcG) proteins are transcriptional inhibitors that remodel chromatin by epigenetic modification and prevent changes of cell characteristics by maintaining transcription states throughout development and into adulthood (Schuettengruber and Cavalli, 2009; Simon and Kingston, 2009; Stojic et al., 2011). In mammals, there are two PcG enhancer of zeste-related genes, enhancer of zeste homologs 1 and 2 (EZH1 and EZH2) (Laible et al., 1997). EZH1 is generally expressed in adults, while EZH2 is expressed during embryonic development (Laible et al., 1997). EZH2 knockout in mouse embryos can lead to embryo post-implantation death (Surface et al., 2010); EZH2 can also recruit transmethylase to its targets in embryonic stem cells (Vire et al., 2006). This indicates that EZH2 is indispensable for mouse embryo development. EZH2, EED, and SUZ12 are the main components of polycomb repressive complex 2 (PRC2), a member of the PcG (Cao et al., 2002). EZH2 is the catalytic subunit of PRC2 and is responsible for the trimethylation of lysine 27 of histone H3 (H3K27me3) (Margueron et al., 2008; Shen et al., 2008) through its SET domain, which is essential for histone lysine methyltransferase activity (Caretti et al., 2004). PRC2 is involved in many biological processes, including cell proliferation, differentiation, stem cell maintenance, embryonic development, and myogenic differentiation (Boyer et al., 2006; Asp et al., 2011; Aloia et al., 2013; Khan et al., 2015). The repression effect of PRC2 on gene expression is mainly through the EZH2 protein (Schuettengruber and Cavalli, 2009; Simon and Kingston, 2009; Margueron and Reinberg, 2011). In skeletal muscle, EZH2 expression is developmentally regulated (Laible et al., 1997); thus, EZH2 may be related to muscle gene expression and differentiation. In proliferating myoblasts, EZH2 and histone deacetylase 1 (HDAC1) are recruited by YY1 to the genomic regions of inactive muscle-specific genes. Upon differentiation, the EZH2-YY1-HDAC1 complex is dissociated, and MyoD and serum response factor are recruited to occupy muscle-specific gene loci (Caretti et al., 2004). Additionally, mice lacking EZH2 in satellite cells will survive with damaged muscle growth (Woodhouse et al., 2013). A previous study showed that EZH2 was required for postnatal muscle growth and adult muscle regeneration (Juan et al., 2011). These observations indicate that EZH2 has important epigenetic roles in skeletal growth and development.

Long non-coding RNAs (lncRNAs) are transcripts greater than 200 nucleotides in length that have no protein coding capacity (Kapranov et al., 2007). lncRNAs have several features including cell type-specific expression patterns (Dinger et al., 2008; Mercer et al., 2008), distinct subcellular localizations (Chen, 2016), regulation of tumorigenesis (Bhan et al., 2017), and evolutionary selection of lncRNA sequences (Ponting et al., 2009), suggesting that lncRNAs may play important roles in various biological processes including skeletal muscle development. For example, lncRNAs such as *SYISL* (Jin et al., 2018), *Malat1* (Chen et al., 2017), *Linc-YY1* (Zhou et al., 2015), *Linc-RAM* (Yu et al., 2017), *Myolinc* (Militello et al., 2018), *MUNC* (Mueller et al., 2015), *Linc-MD1* (Legnini et al., 2014), *Lnc-31* (Dimartino et al., 2018), and *Lnc-SMaRT* (Martone et al., 2020) regulate myogenesis via multiple mechanisms such as chromosome modification, transcription activation, molecular sponge activity, competitive binding, mRNA translation, and protein stability.

Studies have shown that the majority of lncRNAs are located in the nucleus and bind epigenetic modifiers, particularly PRC2 (Zhao et al., 2008; Redrup et al., 2009; Tsai et al., 2010; Grote et al., 2013; Klattenhoff et al., 2013; Kaneko et al., 2014; Mondal et al., 2015; Li X. et al., 2016; Liu et al., 2017; Wang et al., 2018). RNA immunoprecipitation sequencing (RIP-Seq) in mouse embryonic stem cells identified genome-wide PRC2-interacting RNAs, providing evidence for direct RNA–protein interactions, most likely through EZH2 (Zhao et al., 2010). In fetal porcine skeletal muscle, a catalog of fetal porcine long intergenic non-coding RNAs (lincRNAs) has been identified, some of which might interact with EZH2 (Zhao et al., 2015). However, the global identification of EZH2-binding lncRNAs in porcine skeletal muscle and their roles in pig myogenesis are not yet fully understood. In this study, RIP-Seq combined with a lincRNA sequencing (lincRNAseq) method was used to capture EZH2-binding lincRNA transcripts in skeletal muscle from 1-month-old pigs, and 356 novel lincRNAs were identified. A lincRNA *TCONS-00036665* interacting with EZH2 was found to be up-regulated during pig muscle satellite cells (PSCs) and selected for further study. Knockdown and overexpression experiments showed that *TCONS-00036665* promotes cell proliferation but inhibits the differentiation of PSCs. Our study provides a genome-wide view of lincRNAs that specifically associate with EZH2 in pig skeletal muscle and clarifies the function of *TCONS-00036665* in PSCs.

## Materials and Methods

### Animal and Tissue Preparation

Pure Large White female pigs were obtained from the Huazhong Agricultural University farm. All longissimus dorsi muscle samples were collected and maintained at −80°C. C57 mice were purchased from the Hubei Center for Disease Control and housed at Huazhong Agricultural University. All animal experiments were conducted based on the National Research Council Guide for the Care and Use of Laboratory Animals and approved by the Institutional Animal Care and Use Committee at Huazhong Agricultural University.

### RIP-Seq

Longissimus dorsi muscle tissues from 1-month-old pigs were crushed in liquid nitrogen and lysed in ice-cold lysis buffer [50 mM Tris 7.4, 150 mM NaCl, 2 mM ethylenediaminetetraacetic acid (EDTA), 0.1% sodium dodecyl sulfate (SDS), 0.5% NP-40, and 0.5% deoxycholate] with freshly added 1 mM dithiothreitol, 200 U/ml RNase inhibitor, and protease inhibitor cocktail for 30 min on ice. After centrifugation, 300 μl of supernatant was incubated with 10 μg of anti-EZH2 antibody (Bioss bs-3521R) or IgG control antibody overnight at 4°C. The immunoprecipitates were further incubated with protein A Dynabeads for 3 h at 4°C. After applying a magnet and removing the supernatants, the beads were sequentially washed twice with lysis buffer, high-salt buffer (250 mM Tris 7.4, 750 mM NaCl, 10 mM EDTA, 0.1% SDS, 0.5% NP-40, and 0.5 deoxycholate), and PNK buffer (50 mM Tris, 20 mM ethylene glycol tetraacetic acid, and 0.5% NP-40). The immunoprecipitates were eluted from the beads with elution buffer and the RNAs were purified. Then, purified RNAs were reverse-transcribed into complementary DNA (cDNA). The libraries were prepared according to the manufacturer’s instructions and sequenced on an Illumina HiSeq 2000 platform by ABLife, Inc. (Wuhan, China).

### LincRNAseq

Next-generation sequencing was carried out by 1GENE (Hangzhou, China). Briefly, total RNA was extracted from the longissimus dorsi muscle of a 1-month-old pig, ribosomal RNA was removed from total RNA, and the resulting RNA was randomly broken into short segments. Then, the first strand of cDNA was synthesized using random hexamers and the second strand of cDNA was synthesized by adding buffer, dNTPs (except dTTP), dUTP, RNase H, and DNA polymerase I. After purification with a QIAquick PCR kit and elution with EB buffer solution, the ends were repaired, poly(A) tails and sequencing adaptors were added, and the second strand was degraded by uracil-DNA glycosylase enzyme. Then, agarose gel electrophoresis was used to select for fragment size, followed by PCR amplification. Finally, the constructed library was sequenced on the Illumina sequencing platform.

### Identification of EZH2-Binding lincRNAs

The raw data from RIP-Seq and lincRNAseq were cleaned through the following quality control steps: trimming of adaptor sequences and removal of reads containing more than 5% Ns, low-quality reads (based on the percentage of bases with quality score < 20), and short reads. Subsequently, clean reads were aligned to the pig reference genome (Sus_scrofa 10.2) using Tophat2 (Kim et al., 2013) and assembled into transcripts using Cufflinks (Trapnell et al., 2012). The assembled transcripts (gene transfer format files) were compared with the reference transcripts annotated in Sus_scrofa 10.2 using Cuffcompare and yielded a cuffcmp.gtf file with which all novel transcripts were selected for lincRNA identification using the following conditions: transcript length ≥200 bp and located in the intergenic region at least 1 kb from neighboring transcripts.

For all selected transcripts, the coding potentials were evaluated using Coding Potential Calculator (CPC) software (Kong et al., 2007) and cross-species conservation was evaluated by BLAST searches against the pig, mouse, and human expressed sequence tag (EST) databases. The expression levels expressed as fragments per kilobase per million mapped fragments (FPKM) for all characterized transcripts were compared using Cuffdiff among different sequencing datasets.

Then, the lincRNAs were identified using the following screening conditions: FPKM value estimated from the lincRNAseq dataset (lincRNA_FPKM) > 1; FPKM value estimated from the EZH2 RIP-Seq dataset (EZH2_FPKM) > 0; and estimated CPC value ≤ −1. Next, the EZH2-enriched lincRNAs were identified using two different strategies. For any specific lincRNA, if the FPKM value estimated from the IgG RIP-Seq dataset (IgG_FPKM) was >0, we could calculate the ratio of EZH2_FPKM relative to IgG_FPKM as log2[(EZH2_FPKM + 2)/(IgG_FPKM + 2)]. If IgG_FPKM was 0, we could calculate the ratio of EZH2_FPKM relative to lincRNA_FPKM as log2[(EZH2_FPKM + 2)/(lincRNA_FPKM + 2)]. For any transcript, if the ratio was ≥1, it could be regarded an EZH2-enriched lincRNA. Finally, Student’s *t*-test was used to compare the differences in expression levels between different groups.

### Isolation and Culture of PSCs

Satellite cells were isolated from hindlimb muscles of Large White piglets within 1 week, as previously described (Wang et al., 2019a). Briefly, skeletal muscles from piglet hindlimbs were cut into pieces and digested with 0.2% collagenase I (Sigma-Aldrich, St. Louis, MO, United States) in a shaking incubator at 37°C for 2 h. The suspension was then added to an equal volume of RPMI 1640 medium (Gibco, Melbourne, Australia) supplemented with 1% penicillin-streptomycin (Gibco) and filtered through 100-, 200-, and 400-mesh sieves to remove tissue debris. The suspension was centrifuged and the precipitated cells were resuspended in RPMI 1640 proliferation medium supplemented with 20% fetal bovine serum (FBS; Gibco), 1% GlutaMAX (Gibco), 1% non-essential amino acids (Gibco), 0.5% chicken embryo extract (Gemini Bio, West Sacramento, CA, United States), 1% penicillin-streptomycin, and 0.25 μg/100 ml basic fibroblast growth factor (Life Technologies, Carlsbad, CA, United States). The cell suspension was cultured in uncoated plates for 2 h to remove fibroblasts by differential adhesion. Then, the purified satellite cells were transferred to a plate coated with Matrigel (BD Biosciences, San Jose, CA, United States) for proliferating culture in humidified air containing 5% CO_2_ at 37°C in a cell incubator. When the cells reached 60–70% confluence, the RPMI 1640 proliferation medium was replaced with differentiation medium consisting of high-glucose Dulbecco’s modified Eagle’s medium (DMEM; Hyclone Laboratories, San Angelo, TX, United States) supplemented with 2% horse serum (Gibco) and 1% penicillin-streptomycin.

### C2C12 Myoblast Culture

C2C12 myoblasts were obtained from the American Type Culture Collection and cultured in 10% (v/v) FBS in DMEM under humidified air containing 5% CO_2_ at 37°C, and then differentiated at confluence in DMEM with 2% horse serum.

### Rapid Amplification of cDNA Ends (RACE)

The Takara SMARTer RACE cDNA amplification Kit (Clontech, Mountain View, CA, United States) was used for 5’ and 3′ RACE according to the manufacturer’s instructions. The gene-specific primers used for RACE PCR were as follows: 5′ RACE (5′-TGCCTTCTTGCTCATGTTCAGGGCATCG-3′); 3′ RACE (5′-TACTCACAGGAAGCCACCTGGCAAACTG-3′). The PCR products were separated by agarose gel electrophoresis on 1.5% agarose gels, and the bands were purified and cloned into the pMD-18T vector. Positive clones were selected and sequenced by Sangon Biological Technology (Shanghai, China).

### Small Interfering RNA (siRNA) Synthesis and Transfection

siRNA oligos against *TCONS-00036665* were designed using the BLOCK-iT RNAi Designer and synthesized by GenePharma (Shanghai, China). Transfection of siRNA was carried out using Lipofectamine 2000 (Invitrogen, Carlsbad, CA, United States). The *TCONS-00036665* siRNA sequences were as follows: sense 5′-GCUCCAUCUUGCCCUAUAUACTT-3′, antisense 5′-GUAUAUAGGGCAAGAUGGAGCTT-3′.

### Vector Construction, Virus Production, and Infections

The full-length *TCONS-00036665* was obtained by PCR amplification with *TCONS-00036665* full-length forward and reverse primers using PSCs cDNA as a template and then introduced into the lentiviral vector pCDH-CMV-MCS-EF1-copGFP. Then, the recombinant virus plasmid was co-transfected with psPAX2, a lentiviral packaging plasmid, and pMD2.G, a lentiviral envelope plasmid, into 293T cells using DNA transfer reagent (Biomed, Beijing, China) to produce the recombinant lentivirus Lenti-*TCONS-00036665*. At 48 and 72 h after transfection, the virus was harvested and concentrated by ultracentrifugation at 30,000 × *g* for 2.5 h. The concentrated virus was used to infect cells and animals.

For cell infection, an appropriate amount of Lenti-*TCONS-00036665* or Lenti-EV virus was diluted in proliferation medium in the presence of 2 μg/ml of polybrene and the mixture was added to cells.

For animal infection, 6-week-old C57 male mice were intramuscularly injected with an appropriate amount of Lenti-*TCONS-00036665* or Lenti-EV virus in the quadriceps (Qu), tibialis anterior (TA), and gastrocnemius (Gas) muscles of the left or right hind legs, respectively. The injection was carried out once a week for 1 month. After 1 month of injections, mice were sacrificed and the Qu, TA, and Gas muscles were collected for further RNA and protein extraction and histological staining.

### Quantitative Real-Time PCR (qPCR)

Total RNA from isolated PSC, C2C12, or mouse tissue samples was extracted using TRIzol reagent (Invitrogen). cDNA was synthesized using the RevertAid RT Reverse Transcription Kit (Invitrogen). SYBRGreen Real-time PCR Master Mix (Toyobo, Osaka, Japan) was used for qPCR, which was performed in a CFX96/384 Real-Time PCR Detection System (Bio-Rad, Hercules, CA, United States). The 2^–Δ^ ^Δ^ ^*Ct*^ method was used to analyze the relative gene expression data, as described previously (Livak and Schmittgen, 2001). The primers used for qPCR are listed in Supplementary Table S1.

### Western Blotting

Cells and muscle tissue were lysed in RIPA buffer (Beyotime Biotechnology, Jiangsu, China) according to the manufacturer’s instructions. Protein lysates were heated at 95°C for 5 min in 5 × SDS sample buffer, then separated by 10% SDS polyacrylamide gel electrophoresis (30 μg each lane), followed by transfer to a polyvinylidene fluoride membrane (Millipore, Burlington, MA, United States) using a Mini Trans-Blot Cell system (Bio-Rad). The membrane was blocked with 5% non-fat milk for 3 h. The primary antibodies were incubated overnight at 4°C. The membranes were washed and incubated with secondary antibodies for 1 h at 37°C followed by visualization by enhanced chemiluminescence (Bio-Rad). Primary antibodies specific for EZH2 (ab3748, 1:1,000; Abcam, Cambridge, United Kingdom), MyoD (sc-760, 1:1,000; Santa Cruz Biotechnology, Dallas, TX, United States), MyoG (sc-12732, 1:200; Santa Cruz Biotechnology), MyHC (sc-376157, 1:3,00; Santa Cruz Biotechnology), Ki67 (ab16667, 1:1,000; Abcam), p21 (sc-6246, 1:1,000; Santa Cruz Biotechnology), and β-actin (sc-4777, 1:1,000; Santa Cruz Biotechnology), along with goat anti-mouse IgG-HRP (sc-2005, 1:3,000; Santa Cruz Biotechnology) and goat anti-rabbit IgG-HRP (sc-2004, 1:3,000; Santa Cruz Biotechnology) secondary antibodies were used to detect protein expression.

### Cell Proliferation Assays

For flow cytometry, isolated PSCs were immobilized in 70% (v/v) ethanol overnight at −20°C and incubated in 50 mg/ml propidium iodide for 30 min at 4°C. Cell cycles were detected using a FACSCalibur Flow Cytometer (Becton Dickinson, Franklin Lakes, NJ, United States) and the data were analyzed using ModFit software (Verity Software House, Topsham, ME, United States).

For 5-ethynyl-2′-deoxyuridine (EdU) staining, isolated PSCs or C2C12 cells were fixed in 4% paraformaldehyde and permeated in 0.5% Triton X-100. The cells were then stained with EdU using an EDU kit (RiboBio, Guangdong, China) according to the manufacturer’s instructions. Cell nuclei were stained with 4′,6-diamidino-2-phenylindole (DAPI) reagent. Images were captured using an Olympus IX51-A21PH fluorescence microscope (Olympus, Tokyo, Japan) and the percentage of EdU^+^ cells was further analyzed using ImageJ.

### Immunofluorescence Staining of Cells

Cells were washed twice with phosphate-buffered saline (PBS), fixed in 4% paraformaldehyde for 30 min, and permeated in 0.5% Triton X-100 for 30 min. Cells were then blocked with 3% bovine serum albumin for 2 h. Next, cells were incubated with MyHC (1:200) antibody overnight at 4°C. The next day, cells were washed three times with PBS and incubated with a secondary antibody (anti-mouse CY3; Beyotime Biotechnology) for 1 h at 37°C. Then, cell nuclei were stained with DAPI reagent. Images were visualized using an Olympus IX51-A21PH fluorescence microscope. To calculate the cell differentiation index, total and MyHC^+^ nuclei were quantified using ImageJ; the cell differentiation index is represented as the proportion of MyHC^+^ nuclei relative to total nuclei in one field of view.

### Immunofluorescence Staining of Tissue Sections

The Qu, TA, and Gas muscles were fixed in 4% paraformaldehyde and embedded in paraffin, and 4 μm-thick serial sections were obtained. The sections were subjected to dewaxing and antigen repair processes and then immunofluorescence staining was performed as described above for immunofluorescence staining of cells. The antibodies were myosin (M4276, 1:1,000; Sigma-Aldrich) and dystropin (ab15277, 1:200; Abcam). The images were captured using an Olympus DP80 upright Metallurgical Microscope (Olympus), and the cross-section areas of individual myofibers were qualified using ImageJ.

### RNA Pulldown Assay

The full-length *TCONS-00036665* was cloned to the pGEM-3z vector, and the recombinant vector was linearized by single-enzyme digestion. The linearized DNA was labeled with biotin and *in vitro* transcribed using Biotin RNA Labeling Mix and T7 RNA polymerase (Roche, Basel, Switzerland). The obtained RNA was purified with an RNeasy Mini Kit (Qiagen, Hilden, Germany). Total protein was extracted from PSCs using RNA immunoprecipitation (RIP) buffer, then 1 mg of protein was incubated with 3 μg of biotinylated RNA at room temperature for 1 h. Next, each RNA–protein complex was incubated with 40 μl of streptavidin-coupled Dynabeads (Invitrogen) at room temperature for 1 h. Finally, the beads were washed five times with RIP buffer and the pulled down proteins were used for Western blotting.

### Chromatin Immunoprecipitation (ChIP) Assay

Chromatin Immunoprecipitation assays were conducted using a ChIP Assay Kit (P2078; Beyotime) according to the manufacturer’s instructions. Briefly, the cells were fixed with 37% formaldehyde, neutralized with glycine solution, and then washed three times with PBS containing 1 mM phenylmethylsulfonyl fluoride (PMSF). Next, the cells were centrifuged and resuspended in SDS lysis buffer containing 1 mM PMSF. After ultrasonic crushing treatment, the samples were resuspended in ChIP dilution buffer containing 1 mM PMSF. Each ChIP reaction was incubated with 1 μg of antibody against EZH2 (ab3748; Abcam), H3K27me3 (ab6002; Abcam), or IgG (negative control) overnight at 4°C. The next day, 60 μl of protein A + G agarose/salmon sperm DNA was added and the mixture was incubated for 1 h at 4°C. Then, the pulled down DNA was eluted and purified. Fold enrichment was quantified by qPCR. All promoter primers are listed in Supplementary Table S2.

### Data Availability

The raw data of RIP-Seq and lincRNAseq generated in this study have been deposited to the Gene Expression Omnibus (GEO) database with the accession number GSE155260.

### Statistical Analysis

Data are presented as means ± standard deviation. Statistical analyses between different groups were determined by Student’s *t*-test. For all analyses, a *P* < 0.05 was considered to indicate statistical significance.

## Results

### RIP-Seq Identified EZH2-Binding lincRNAs

**FIGURE 1:**
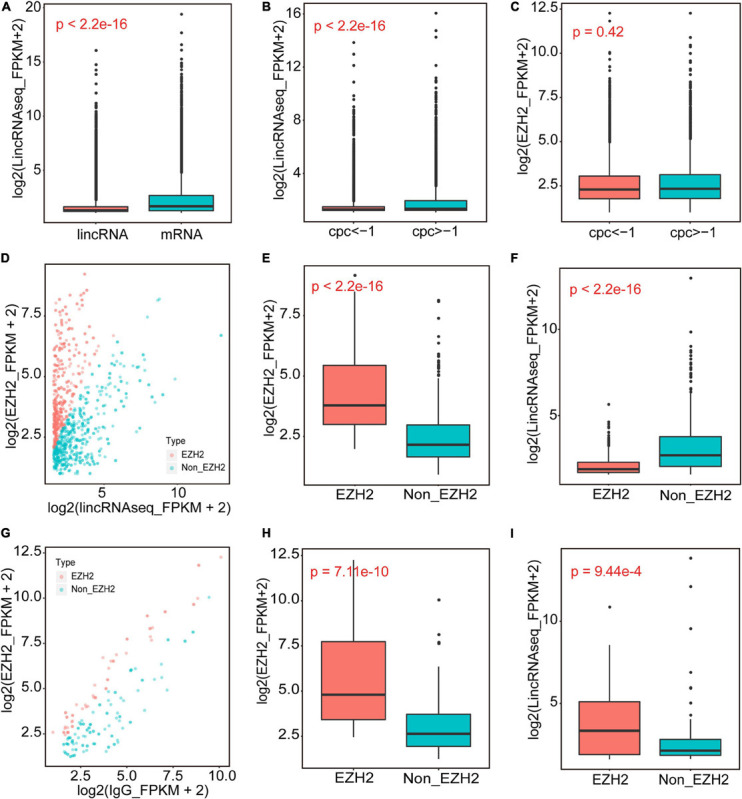
The identification of EZH2-binding novel lincRNAs. **(A)** The expression levels of novel lincRNAs and mRNAs annotated in Ensembl. **(B,C)** The expression levels of novel lincRNAs with different CPC groups. **(D–F)** The EZH2-enriched lincRNAs identified when IgG_FPKM was 0. Volcano plots representing the distribution of log2(lincRNAseq_FPKM + 2) (*X*-axis) and log2(EZH2_FPKM + 2) (*Y*-axis); the red dots represent EZH2-enriched lincRNAs, and the blue dots represent Non-EZH2-enriched lincRNAs **(D)**. The EZH2_FPKM values of EZH2-enriched lincRNAs group and Non-EZH2-enriched lincRNAs group **(E)**. The lincRNA expression levels of EZH2-enriched lincRNAs group and Non-EZH2-enriched lincRNAs group **(F)**. **(G–I)** The EZH2-enriched lincRNAs identified when IgG_FPKM was >0. Volcano plots representing the distribution of log2(IgG_FPKM + 2) (*X*-axis) and log2(EZH2_FPKM + 2) (*Y*-axis) **(G)**. The red dots represent EZH2-enriched lincRNAs, and the blue dots represent Non-EZH2-enriched lincRNAs. The EZH2_FPKM values of EZH2-enriched lincRNAs group and Non-EZH2-enriched lincRNAs group **(H)**. The lincRNA expression levels of EZH2-enriched lincRNAs group and Non-EZH2-enriched lincRNAs group **(I)**.

**FIGURE 2:**
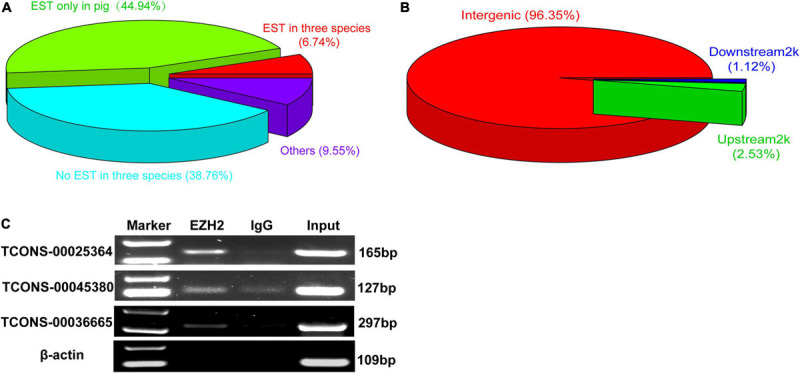
The verification of EZH2-binding novel lincRNAs. **(A)** The EST information of those novel lincRNA by EZH2 RIP. **(B)** The location of those novel lincRNA by EZH2 RIP. **(C)** RIP-PCR results showed that three lincRNAs, *TCONS-00025364*, *TCONS-00045380*, and *TCONS-00036665*, could bind to EZH2.

We performed RIP assays with skeletal muscle of a 1-month-old pig. After incubation with EZH2 or IgG antibody, the samples were centrifuged, and supernatant and precipitate were collected separately for Western blotting. Specific bands could be detected for precipitates of the EZH2 antibody but not the IgG antibody (Supplementary Figure S1), indicating that EZH2 antibody enrichment was successful and its precipitates could be used for RIP-Seq. The pulled down RNAs were purified for RIP sequencing on an Illumina HiSeq 2000 system. To provide a potential control, we also performed lincRNAseq with the same sample. To identify EZH2-enriched lincRNAs, we conducted several filtering steps (Supplementary Figure S2). First, we set the lincRNA_FPKM value to >0.1 and obtained 21,455 novel expressed transcripts that displayed significantly lower expression levels than the mRNA annotated in the reference genome (Figure 1A). Second, we added the condition of an EZH2_FPKM value >0 and obtained 9,709 transcripts, of which 5,963 had a CPC value ≤ -1. The transcripts with a CPC value > −1 displayed significantly higher expression levels than those of transcripts with a CPC < -1 (Figure 1B), whereas their enrichment levels in precipitates of the EZH2 antibody showed no significant difference (Figure 1C). Third, based on the above screening, we further set the lincRNA_FPKM value to >1 to remove some lowly expressed lincRNAs. Furthermore, we performed enrichment analysis to further identify EZH2-enriched lincRNAs. Based on the ratio of EZH2_FPKM to lincRNA_FPKM, we identified a total of 496 EZH2-binding lincRNAs and 598 non-EZH2-binding lincRNAs (Figure 1D). The EZH2-binding lincRNAs displayed significantly higher EZH2_FPKM values (Figure 1E) but lower expression levels (Figure 1F) than the non-EZH2-binding lincRNAs. Based on the ratio of EZH2_FPKM to IgG_FPKM, we identified a total of 58 EZH2-binding lincRNAs and 116 non-EZH2-binding lincRNAs (Figure 1G). The EZH2-binding lincRNAs displayed significantly higher EZH2_FPKM values (Figure 1H) and expression levels (Figure 1I) than the non-EZH2-binding lincRNAs. After manually removing the repetitive lincRNAs, we finally obtained 356 EZH2-binding lincRNAs (Supplementary Table S3), of which 24 have ESTs in pigs, mice, and humans, 138 have no ESTs in the three species, and 160 lincRNAs have ESTs only in pigs (Figure 2A). Not surprisingly, almost all EZH2-binding lincRNAs were in intergenic regions (96.75%) (Figure 2B).

### Verification of EZH2-Binding lincRNAs and Molecular Characterization of *TCONS-00036665*

**FIGURE 3:**
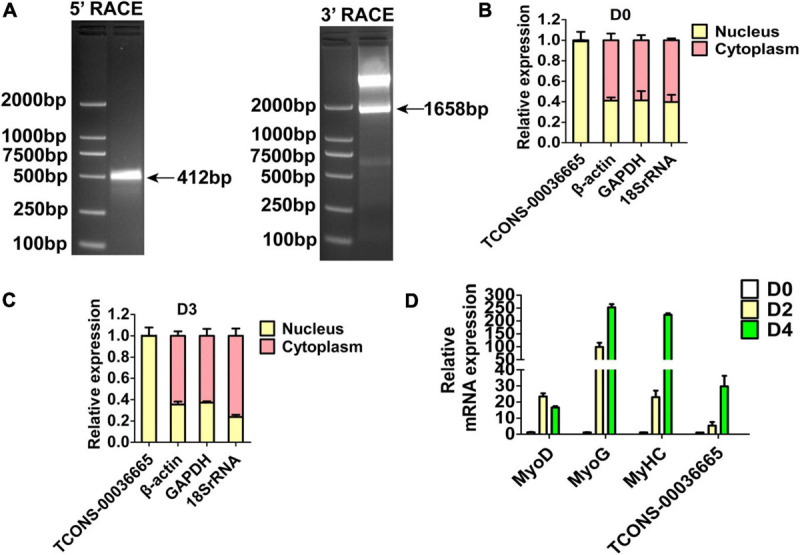
The molecular characterization of *TCONS-00036665*. **(A)** Detection of full-length *TCONS-00036665* by 5′ RACE and 3′ RACE. **(B,C)** Detection of the distribution of *TCONS-00036665* in the cytoplasm and nucleus of proliferating pig primary myoblasts **(B)** and differentiated 3 days **(C)**. **(D)** qPCR results showed that the expression of *TCONS-00036665* was induced during PSC differentiation. The relative RNA levels were normalized to those of the control β*-actin*. Error bars represent the mean ± SD of three biological replicates.

Three lincRNAs, *TCONS-00025364*, *TCONS-00045380*, and *TCONS-00036665*, were selected and verified to bind EZH2 by RIP-PCR experiments (Figure 2C). Among the three lincRNAs, the sequences of *TCONS-00036665* aligned with the pig *EF397601* transcript, which shows similarity to human and mouse *NEAT1* gene when aligned it to the National Center for Biotechnology Information (NCBI) database. *EF397601* was first identified in Tongcheng pig with a full length of 3409 bp and up-regulated in prenatal skeletal muscle (Ren et al., 2009), indicating that it may be involved in skeletal muscle development. Therefore, we selected *TCONS-00036665* for further study. To examine the function of *TCONS-00036665*, the full-length sequence of *TCONS-00036665* was obtained using rapid amplification of cDNA ends (RACE). RACE results showed that *TCONS-00036665* was a 3,450 bp polyadenylated transcript (Figure 3A and Supplementary Table S4); it has been deposited in GenBank (accession number MT248265). Next, cell-fractionation assays were performed in PSCs at the proliferation and differentiation stages for 3 days. The results demonstrated that *TCONS-00036665* was distributed in the nuclei of proliferating and differentiated PSCs (Figures 3B,C). Finally, we detected the expression of *TCONS-00036665* during PSC differentiation and found that the expression of *TCONS-00036665* increased during PCS differentiation at 0, 2, and 4 days (Figure 3D). All of these characteristics of *TCONS-00036665* imply that it may play a role in PSC proliferation and differentiation.

### *TCONS-00036665* Promotes PSC Proliferation and Inhibits PSC Differentiation

**FIGURE 4:**
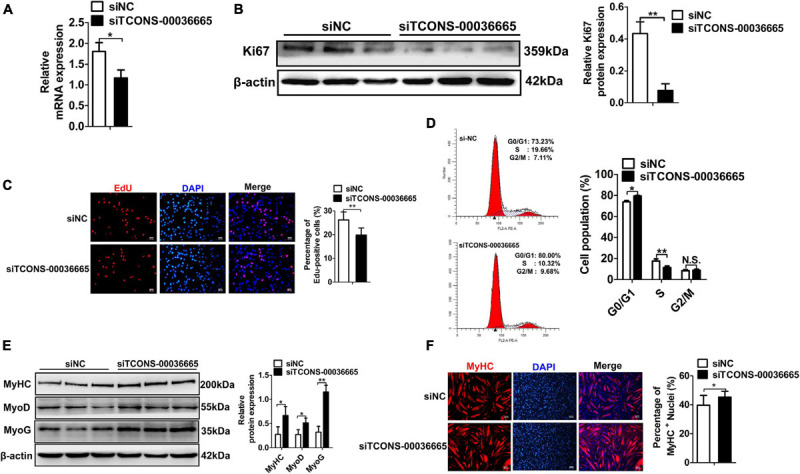
Knockdown of *TCONS-00036665* inhibits PSC proliferation but promotes PSC differentiation. **(A)** qPCR results showed that *TCONS-00036665* expression was significantly reduced. **(B)** Western blotting results showed that the proliferating marker gene Ki67 protein expression was significantly reduced after *TCONS-00036665* knockdown, and relative Ki67 protein expression was quantified by ImageJ software. **(C)** EdU staining results showing that cell proliferation ability was remarkably inhibited by *TCONS-00036665* knockdown. **(D)** Flow cytometry analysis results showing that knockdown of *TCONS-00036665* remarkably decreased the proportion of S phase, but increased the proportion of G1 phase. **(E)** Western blotting results showing that the protein expression of myogenic marker genes MyoD, MyoG, and MyHC was significantly increased by *TCONS-00036665* knockdown, and the relative MyoD, MyoG, and MyHC protein expression was quantified by ImageJ software. **(F)** MyHC immunofluorescence staining results showing that the proportion of MyHC^+^ was significantly enhanced by *TCONS-00036665* knockdown. The relative RNA and protein levels were normalized to those of the control β-actin. Error bars represent the mean ± SD of three biological replicates. *p < 0.05, **p < 0.01, N.S. indicates not significant.

To investigate the role of *TCONS-00036665* in PSC proliferation, we knocked down *TCONS-00036665* expression with siRNA (Figure 4A). *TCONS-00036665* knockdown significantly decreased protein expression of the key proliferation marker Ki67 (Figure 4B). Then, EdU staining and flow cytometry were used to detect the role of *TCONS-00036665* in PSC proliferation. EdU staining revealed that knockdown of *TCONS-00036665* significantly reduced the proportion of EdU^+^ cells compared to control (Figure 4C). Flow cytometry showed that *TCONS-00036665* knockdown significantly decreased the percentage of S-phase cells (Figure 4D). These observations demonstrated that *TCONS-00036665* knockdown repressed PSC proliferation. To verify the effect of *TCONS-00036665* knockdown on PSC differentiation, Western blotting and immunofluorescence staining were used to detect expression of myogenic marker genes. Western blotting revealed that the protein expression of MyoD, MyoG, and MyHC was significantly increased by *TCONS-00036665* knockdown (Figure 4E). Immunofluorescence staining of MyHC showed that *TCONS-00036665* knockdown remarkably enhanced PSC differentiation (Figure 4F). The above results indicated that *TCONS-00036665* knockdown inhibits PSC proliferation and accelerates PSC differentiation.

**FIGURE 5:**
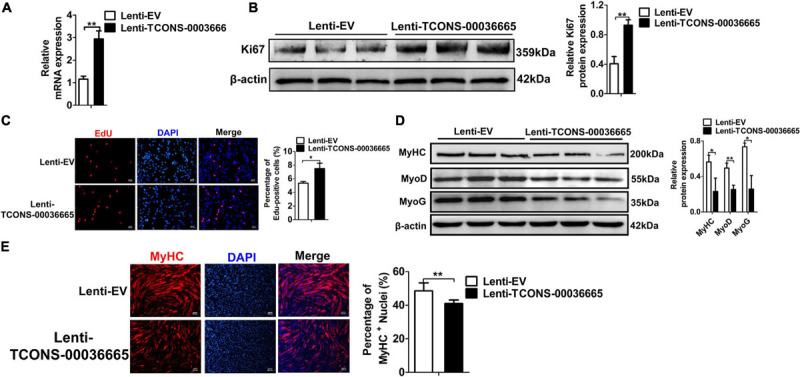
*TCONS-00036665* overexpression promotes PSC proliferation but inhibits PSC differentiation. **(A)** qPCR results showing that *TCONS-00036665* expression was significantly increased. **(B)** Western blotting results showed that Ki67 protein expression was significantly enhanced by *TCONS-00036665* overexpression, and relative Ki67 protein expression was quantified by ImageJ software. **(C)** EdU staining results showing that *TCONS-00036665* overexpression remarkably enhanced cell proliferation ability. **(D)** Western blotting results showing that the protein expression of MyoD, MyoG, and MyHC was significantly inhibited by *TCONS-00036665* overexpression, and relative MyoD, MyoG, and MyHC protein expression was quantified by ImageJ software. **(E)** MyHC immunofluorescence staining results showing that the proportion of MyHC^+^ was significantly decreased by *TCONS-00036665* overexpression. The relative RNA and protein levels were normalized to those of the control β-actin. Error bars represent the mean ± SD of three biological replicates. *p < 0.05, **p < 0.01.

To further verify the role of *TCONS-00036665* in the proliferation and differentiation of PSCs, *TCONS-00036665* was overexpressed in PSCs (Figure 5A). Overexpression of *TCONS-00036665* significantly increased the protein expression of Ki67 (Figure 5B) and the percentage of EdU^+^ cells (Figure 5C), indicating that overexpression of *TCONS-00036665* promoted PSC proliferation. *TCONS-00036665* overexpression remarkably reduced protein expression of MyoD, MyoG, and MyHC (Figure 5D) as well as the proportion of MyHC^+^ cells (Figure 5E), indicating that *TCONS-00036665* overexpression inhibited the differentiation of PSCs. These results all demonstrate that *TCONS-00036665* can promote the proliferation and inhibit the differentiation of PSCs.

### Overexpression of *TCONS-00036665* Promotes Proliferation but Inhibits Differentiation of Mouse C2C12 Myoblasts

**FIGURE 6:**
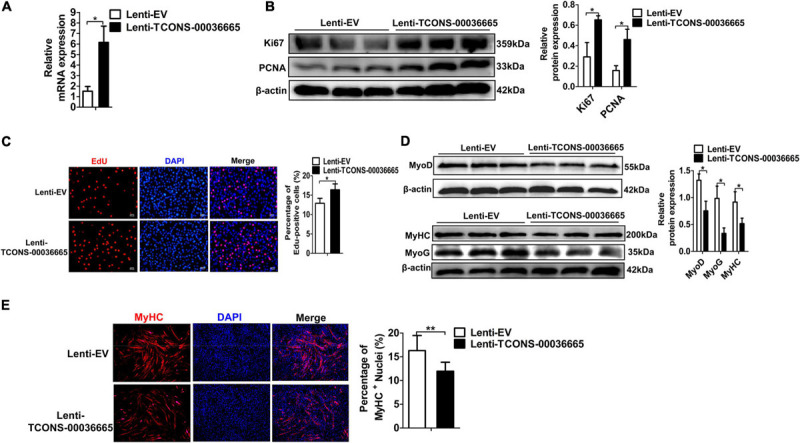
*TCONS-00036665* overexpression promotes the proliferation but inhibits the differentiation of mouse C2C12 myoblasts. **(A)** qPCR results showed that *TCONS-00036665* expression was significantly increased in C2C12 cells. **(B)** Western blotting results showed that *TCONS-00036665* overexpression significantly increased Ki67 and PCNA protein expression in C2C12 cells, and the relative Ki67 and PCNA protein expression was quantified by ImageJ software. **(C)** EdU staining results showed that *TCONS-00036665* overexpression remarkably promoted C2C12 cell proliferation ability. **(D)** Western blotting results showed that MyoD, MyoG, and MyHC protein expression was significantly inhibited by *TCONS-00036665* overexpression in C2C12 cells, and the relative MyoD, MyoG, and MyHC protein expression was quantified by ImageJ software. **(E)** MyHC immunofluorescence staining results showing that *TCONS-00036665* overexpression significantly decreased MyHC^+^ proportion of C2C12 myotubes. The relative RNA and protein levels were normalized to those of the control β-actin. Error bars represent the mean ± SD of three biological replicates. *p < 0.05, **p < 0.01.

To verify whether *TCONS-00036665* function is conserved between pigs and mice, we overexpressed *TCONS-00036665* in mouse C2C12 myoblasts (Figure 6A) and examined its roles in C2C12 proliferation and differentiation. Western blot analysis of proliferating C2C12 cells showed that overexpression of *TCONS-00036665* increased the protein expression of proliferation-related genes Ki67 and PCNA (Figure 6B). EdU staining revealed that the percentage of EdU^+^ cells was significantly increased after *TCONS-00036665* overexpression (Figure 6C). These results indicated that *TCONS-00036665* overexpression enhanced the proliferation ability of C2C12 cells. To investigate the effect of *TCONS-00036665* on C2C12 differentiation, the protein expression of differentiation marker genes was analyzed by Western blotting and the expression of MyoD, MyoG, and MyHC proteins was decreased by *TCONS-00036665* overexpression (Figure 6D). Immunofluorescence staining of MyHC revealed that *TCONS-00036665* overexpression remarkably inhibited C2C12 differentiation (Figure 6E), suggesting that *TCONS-00036665* overexpression suppressed C2C12 differentiation. These observations indicate that *TCONS-00036665* overexpression promotes the proliferation but inhibits the differentiation of mouse C2C12 myoblasts, indicating the conserved function of *TCONS-00036665* during pig and mouse myogenesis.

### Overexpression of *TCONS-00036665* Inhibits Mouse Skeletal Muscle Development *in vivo*

**FIGURE 7:**
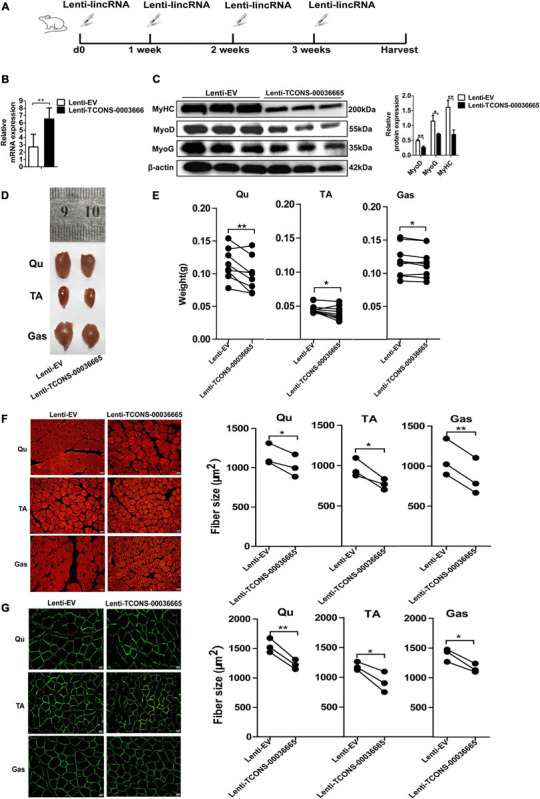
*TCONS-00036665* overexpression inhibits mouse skeletal muscle development *in vivo*. **(A)** The injection scheme of lenti-*TCONS-00036665* or lenti-EV particles into the hindlimb muscles of C57 mice. The injection was performed every 1 week, and the mice were sacrificed after a month injection. **(B)** qPCR results showing that *TCONS-00036665* expression was significantly increased. **(C)** Western blotting results showing that the protein expression of MyoD, MyoG, and MyHC was significantly inhibited by *TCONS-00036665* overexpression, and the relative MyoD, MyoG, and MyHC protein expression was quantified by ImageJ software. **(D)** Representative pictures of quadriceps (Qu), tibialis anterior (TA), and gastrocnemius (Gas) muscles from the right or left hindlimbs of mice. **(E)** The statistical analysis of the weight of Qu, TA, and Gas muscles from the right or left hindlimbs of nine injected mice; the results showed that overexpression of *TCONS-00036665* decreased the weight of each muscle. **(F,G)** Representative pictures of myosin **(F)** and dystrophin **(G)** immunofluorescence staining of the cross section of Qu, TA, and Gas muscles in mice showing that *TCONS-00036665* overexpression remarkably reduced the cross-sectional area of each muscle. The cross-sectional area of each muscle was quantified by ImageJ. The relative RNA and protein levels were normalized to those of the control β-actin. Error bars represent the mean ± SD of three biological replicates. *p < 0.05, **p < 0.01.

To study the function of *TCONS-00036665* in muscle development, *TCONS-00036665* was packed with lentivirus to produce Lenti-*TCONS-00036665* and injected into the muscles of mice. Lenti-*TCONS-00036665* and Lenti-EV were injected into the right and left hindlimbs of 6-week-old C57 mice, respectively (Figure 7A). Reverse transcription qPCR showed that *TCONS-00036665* expression was significantly increased by Lenti-*TCONS-00036665* injection (Figure 7B). The injection of Lenti-*TCONS-00036665* led to a significant reduction in the protein expression of MyoD, MyoG, and MyHC compared to Lenti-EV injection (Figure 7C). The volume and weight of Qu, Gas, and TA muscles were reduced by *TCONS-00036665* overexpression (Figures 7D,E). Immunofluorescence staining with myosin and dystropin revealed that the cross-sectional areas of Qu, Gas, and TA muscles were significantly decreased after *TCONS-00036665* overexpression (Figures 7F,G). These results demonstrate that *TCONS-00036665* overexpression can repress mouse skeletal muscle development.

### *TCONS-00036665* Regulates Myogenesis Through EZH2

**FIGURE 8:**
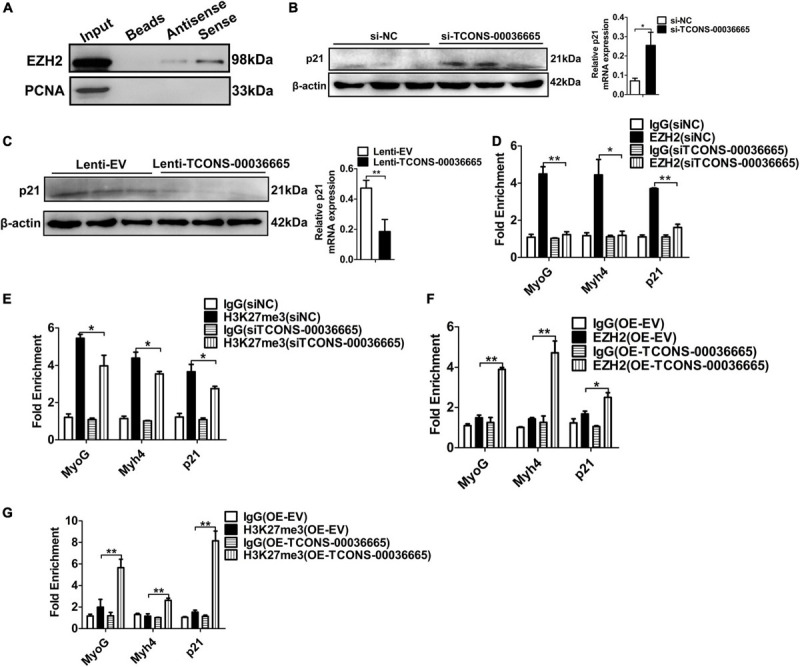
*TCONS-00036665* regulates myogenesis through EZH2. **(A)** Biotin-labeled full-length *TCONS-00036665* was used to pull down EZH2. Western blotting result showed that *TCONS-00036665* could interact with EZH2. PCNA is a nucleoprotein used as negative controls. **(B,C)** Western blotting results showed that p21 protein expression was increased by *TCONS-00036665* knockdown **(B)** but decreased by *TCONS-00036665* overexpression **(C)**. Relative p21 protein expression was quantified by ImageJ software. **(D,E)** ChIP-qPCR results showed that the enrichment of EZH2 (D) and H3K27me3 **(E)** proteins on the promoters of *p21*, *MyoG*, and *Myh4* were significantly reduced after *TCONS-00036665* knockdown in PSCs. **(F,G)** ChIP-qPCR results showed that the enrichment of EZH2 **(F)** and H3K27me3 **(G)** proteins on the promoters of *p21*, *MyoG*, and *Myh4* were significantly enhanced after *TCONS-00036665* overexpression in PSCs. The relative protein levels were normalized to those of the control β-actin. Error bars represent the mean ± SD of three biological replicates. *p < 0.05, **p < 0.01.

To verify whether *TCONS-00036665* regulates myogenesis by interacting with EZH2, we first confirmed the interaction between *TCONS-00036665* and EZH2 by RNA pulldown assay (Figure 8A). Then, Western blotting revealed that *TCONS-00036665* knockdown significantly enhanced p21 protein expression (Figure 8B) while *TCONS-00036665* overexpression repressed p21 protein expression (Figure 8C), indicating that *TCONS-00036665* can inhibit p21 protein expression. Furthermore, ChIP assay revealed that knockdown of *TCONS-00036665* significantly decreased the enrichment of EZH2 and H3K27me3 on *p21*, *MyoG*, and *Myh4* promoters (Figures 8D,E), while EZH2 and H3K27me3 enrichment on the promoters of *p21*, *MyoG*, and *Myh4* genes was significantly increased by *TCONS-00036665* overexpression (Figures 8F,G). These results indicate that *TCONS-00036665* can recruit EZH2 to the promoters of *p21*, *MyoG*, and *Myh4*, inhibiting their gene expression and thus promoting PSC proliferation and repressing PSC differentiation.

## Discussion

EZH2-mediated epigenetic repression plays an important role in skeletal muscle specification (Stojic et al., 2011; Dudakovic et al., 2015; Lui et al., 2016; Adhikari and Davie, 2018). EZH2 represses gene expression by catalyzing H3K27me3 on target gene promoters, which serves as an epigenetic signal for chromatin condensation and transcriptional repression (Lui et al., 2016). Recent studies have reported that EZH2 function may be mediated by other factors, such as lncRNAs. For example, lncRNA *HERES* regulates canonical and non-canonical Wnt signaling pathways through interacting with EZH2 in esophageal squamous cell carcinoma (You et al., 2019). lncRNA *ANCR* regulates breast cancer progression and metastasis by reducing the stability of EZH2 (Li et al., 2017). Our previous study also found that lncRNA *SYISL* and *Neat1* promote myogenic proliferation but repress myogenic differentiation through interaction with PRC2 (Jin et al., 2018; Wang et al., 2019b). Despite these reports, little is known about EZH2-interacting lincRNAs in pig skeletal muscle. Using unbiased RIP-seq combined with lincRNAseq screening for EZH2-interacting lincRNAs in pig skeletal muscle tissue, we identified 356 new lincRNA transcripts binding to EZH2, which provides a database for the study of the EZH2 regulatory network in pigs.

Skeletal muscle growth and development depend on myoblast proliferation and differentiation and determine the quality and quantity of meat production. Elucidating the regulatory network of skeletal muscle growth and development will be helpful for the improvement of agricultural animal meat traits. In the recent years, owing to the development of RNA-Seq and other high-throughput technologies, tens of thousands of lncRNAs have been identified in the skeletal muscle of agricultural animal. However, only a very small part of lncRNAs have been functionally studied. In particular, lncRNAs, like *lncRNA-Six1* (Ma et al., 2018) and *lncIRS1* (Li et al., 2019), were found to regulate chicken skeletal muscle development. LncRNAs, like *lncMD* (Sun et al., 2016), *Lnc133b* (Jin et al., 2017), and *MDNCR* (Li et al., 2018), were found to regulate bovine skeletal muscle development. *Lnc-SEMT* has a role in regulating sheep skeletal muscle development (Wei et al., 2018). In this study, a lincRNA *TCONS-00036665* was functionally characterized. We found that *TCONS-00036665* was specifically localized in the nucleus and induced during PSCs differentiation. Follow-up studies demonstrated that *TCONS-00036665* can promote PSC proliferation but repress differentiation. Furthermore, overexpression of *TCONS-00036665* in mouse C2C12 myoblasts and mouse leg muscle also inhibited C2C12 differentiation and muscle growth. Therefore, we concluded that *TCONS-00036665* plays an important role in pig skeletal muscle development.

The function of lncRNAs is associated with their subcellular localization; lncRNAs that exhibit nuclear localization patterns are important for nuclear function regulation (Chen, 2016). These lncRNAs execute their regulatory roles by multiple mechanisms; for example, they can accumulate to their transcriptional sites and influence the expression of neighboring genes or recruit transcription factors or chromatin-modifying complexes to target gene promoter (Chen, 2016). The subcellular localization results of *TCONS-00036665* showed that it is specifically located in the nucleus, indicating that it may play a role at the transcriptional level. So, we firstly consider that *TCONS-00036665* regulates myogenesis through the epigenetic modifier EZH2. As expected, the interaction between *TCONS-00036665* and EZH2 was confirmed by our RIP and RNA pulldown assays. Our ChIP assays also demonstrated that *TCONS-00036665* can increase the enrichment of EZH2 and H3K27me3 on corresponding target gene promoters. These results confirmed our conjecture that *TCONS-00036665* promotes PSC proliferation but inhibits PSC differentiation by recruiting EZH2 to target gene promoters. LncRNAs may bind to RNA binding proteins (RNPs) through a core binding region; for example, *Linc-YY1* regulates myogenesis by binding to YY1 with its middle part (386-851nt) (Zhou et al., 2015). LncRNA *HOXC-AS3* interacts with YBX1 to mediate gastric cancer tumorigenesis, and a 115 nucleotide region of *HOXC-AS3* are responsible for its binding ability to YBX1 (Zhang et al., 2018). LncRNA *CPR* recruits DNMT3A to Mcm3 promoter and represses cardiomyocyte cell proliferation by binding to DNMT3A with a 244 nucleotide region at the 3′ end (Ponnusamy et al., 2019). Whether *TCONS-00036665* interacts with EZH2 through a core binding region is unknown, which needs to be further study.

In this study, we found that *TCONS-00036665* serves as a scaffold to link proteins to target gene promotes. However, the mechanism of *TCONS-00036665* recruitment to gene promoter remains unclear. Previous studies have proposed two opposing types of RNA–DNA interaction, one of which described that the RNA molecule interacts with one of the two DNA strands by base-pairing to substitute the second strand to form an “R loop” (Skourti-Stathaki and Proudfoot, 2014). The other described that the DNA remains paired and the RNA is wound within the major groove of DNA and forms triplex base pairing between RNA and DNA bases (Li Y. et al., 2016). Furthermore, other studies also reported that proteins may be involved in the interaction and maintenance of RNA–DNA hybridization. For example, the ubiquitous dsRNA-binding protein ILF2 and ILF3 were found to be associated with RNA–DNA hybrids (Nadel et al., 2015), suggesting that it may play a role in RNA–DNA interaction. Thus, we speculated that the recruitment of *TCONS-00036665* to different promoters may be caused by forming an “R loop” or triplex base pairing, and RNA-binding proteins or other factors may also affect the recruitment of *TCONS-00036665* to different promoters.

In summary, using RIP-Seq and RNA-Seq, we identified a total of 356 novel EZH2-binding lincRNAs in pig skeletal muscle. A lincRNA, *TCONS-00036665*, was found to regulate myogenesis and muscle development by increasing the enrichment of EZH2 and H3K27me3 on *p21*, *MyoG*, and *Myh4* promoters.

## Data Availability Statement

The datasets presented in this study can be found in online repositories. The names of the repository/repositories and accession number(s) can be found below: https://www.ncbi.nlm.nih.gov/geo/, GSE155260.

## Ethics Statement

The animal study was reviewed and approved by the Institutional Animal Care and Use Committee at Huazhong Agricultural University.

## Author Contributions

SW performed all experiments, interpreted results, and wrote the manuscript. XX conducted the bioinformatics analysis of RIP-seq and revised the primary version of manuscript. YL performed the data analysis of RIP-seq. JJ performed nuclear and cytoplasmic RNA fractionation and isolated pig satellite cells. FZ collected tissue samples for RIP-seq. WB, YG, and JZ isolated pig satellite cells. HZ provided support and suggestions for histology staining. BZ and ZX conceived the project, revised the manuscript, and approved the final version of the manuscript. All authors contributed to the article and approved the submitted version.

## Conflict of Interest

The authors declare that the research was conducted in the absence of any commercial or financial relationships that could be construed as a potential conflict of interest.
