# Supplementary material for: RIP-Seq of EZH2 Identifies TCONS-00036665 as a Regulator of Myogenesis in Pigs
Source: Front Cell Dev Biol. 2021 Jan 12;8:618617. doi: 10.3389/fcell.2020.618617 (PMC7835406; doi:10.3389/fcell.2020.618617)
Supplement: Supplementary file 1 [file Table_1.DOCX]

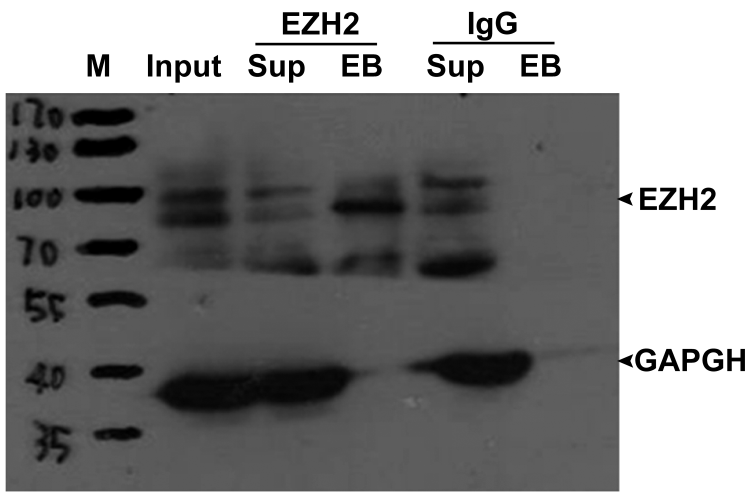


**Figure S1.** **Western blot analysis of EZH2 protein in EZH2 antibody and IgG control sample after RIP.** After incubated with the EZH2 or IgG antibody, the lysates were centrifuged, and the supernatant (Sup) and precipitate (EB) were separated and performed western blotting experiment. The result showed that the majority of the EZH2 protein was detected in the precipitates of the EZH2 antibody, and GAPDH protein could not detect in the precipitates of the EZH2 antibody, which menas the EZH2 antibody can specific bind to EZH2 protein. Meanwhile, EZH2 protein was detected in the supernatant of the IgG antibody, but not the precipitates, indicating that the IgG antibody did not bind to EZH2 nonspecificly.


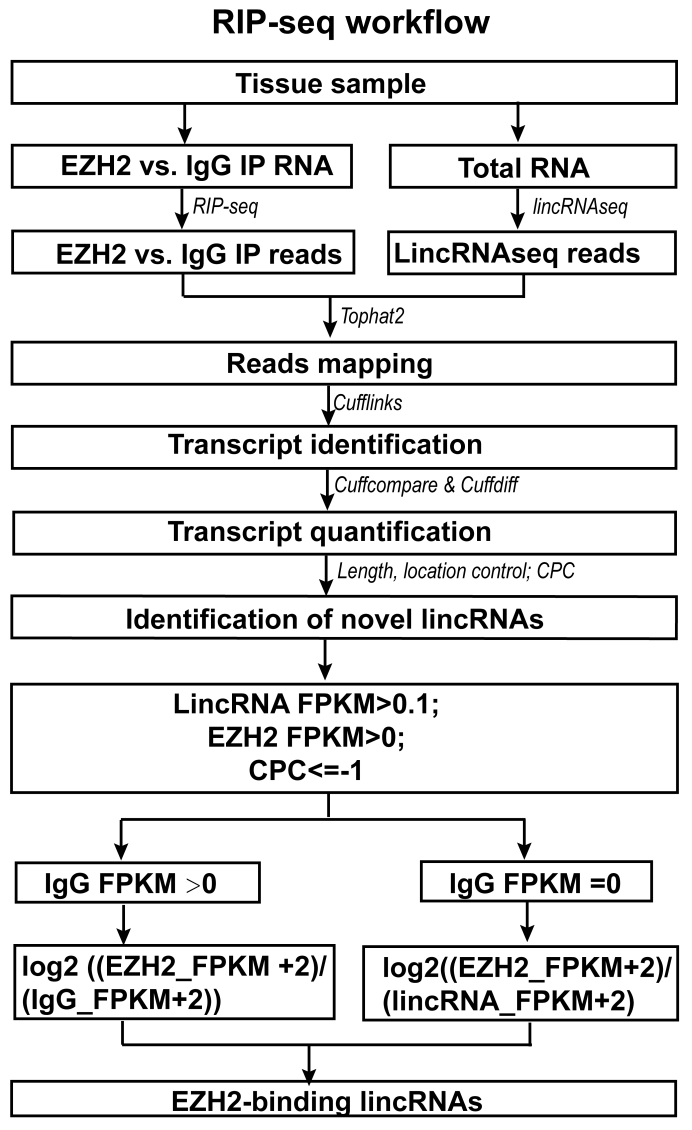


**Figure S2**. Outline of RIP-seq combined with lincRNA-seq analysis pipeline.
