# Supplementary material for: RIP-Seq of EZH2 Identifies TCONS-00036665 as a Regulator of Myogenesis in Pigs
Source: Front Cell Dev Biol. 2021 Jan 12;8:618617. doi: 10.3389/fcell.2020.618617 (PMC7835406; doi:10.3389/fcell.2020.618617)
Supplement: Supplementary file 2 [file Table_2.DOCX]

**Table S1. Primers used for PCR or qPCR**

| **Gene or Primer name** | **Primer sequence(5’-3’)** |
| --- | --- |
| TCONS_00025364 | F:CTCCCCACTGTCTTGCTTCA |
|  | R:GACCCAGTGACCTTGGAGTG |
| TCONS_00045380 | F:ACCCCGAAAACAGACGAGC |
|  | R:CGGACAACCAGCTATCACCA |
| TCONS_00036665(for PCR) | F:GCCGTCGATGCCCTGAACATG |
|  | R:ACGCAGTCCCTAGACCCCCCT |
| TCONS_00036665(for qPCR) | F:CCCTTGGGAGTTACGTCTTCT |
|  | R:CATGCCCAAGTCACAGGTTAAGA |
| MyoD | F:TGCGTATTCTCAACCCCTTC |
|  | R:AGTATGCAAGGGTGGAGTGG |
| MyoG | F:AGGCTACGAGCGGACTGA |
|  | R:GCAGGGTGCTCCTCTTCA |
| MyHC | F:AAGGCATCATCAAGGACACTC |
|  | R:TGCGGCAGGTTGGCTCT |
| β-actin | F:ACGGGACATCAAGGAGAAGC |
|  | R:GGCACTGTGTTGGCGTAGAG |
| GAPDH | F:ACCCAGAAGACTGTGGATGG |
|  | R:AAGCAGGGATGATGTTCTGG |
| 18S RNA | F:GGCTACCACATCCAAGGAAG |
|  | R:TCCAATGGATCCTCGCGGAA |
