# Supplementary material for: RIP-Seq of EZH2 Identifies TCONS-00036665 as a Regulator of Myogenesis in Pigs
Source: Front Cell Dev Biol. 2021 Jan 12;8:618617. doi: 10.3389/fcell.2020.618617 (PMC7835406; doi:10.3389/fcell.2020.618617)
Supplement: Supplementary file 3 [file Table_3.DOCX]

**Table S2. Primers used for ChIP**

| **Gene or Primer name** | **Primer sequence(5’-3’)** |
| --- | --- |
| MyoG | F:CCTTGGGCATCCCCGTAATCC |
|  | R:GGGCTTCTCAGTCCATAGTCAC |
| Myh4 | F:TGCTGCTATGTCAAACCAGGAT |
|  | R:CAGGTCTGGTGTTGGCCTTA |
| p21 | F:CCTTGGGCATCCCCGTAATCC |
|  | R:ATTCGAGTCAGGCCAGGATTG |
