# Supplementary material for: RIP-Seq of EZH2 Identifies TCONS-00036665 as a Regulator of Myogenesis in Pigs
Source: Front Cell Dev Biol. 2021 Jan 12;8:618617. doi: 10.3389/fcell.2020.618617 (PMC7835406; doi:10.3389/fcell.2020.618617)
Supplement: Supplementary file 5 [file Table_5.DOCX]

**Table S4. The full length of TCONS-00036665**

GGGCCTGGGCTGAGTTGTGGGGGAGGAGGTGGCAGTTTAGGGCCTCAGGGGACATATGGGGGCGGTGGCAGAGTCAGGCGATACTGGCGGGCTGGGGGTGGGAGTGTGCCTGCTGGAGGCAGGTTAGAGGGGAGACTAGACCTTAGGGAAGTCTTGCAGGGCAGAGCTGCCTCGAAAGGGCCCGCAGGAACCCGGGGAGAGGAGCACACTCCAGGCCTGGGTGTCGCTGCTGCCCGCTGGAGAAGGGGAACTTGACCCCTGGGAGGGGGCCGATTCCGCGTAGGCTGGGCGGCCCCCTGTGCCCTGCTTTGTCTGACGGAGGGAGGCAGAGACTGGAGGAGGCTGAGGGTCTCGCCACCCTTGCTGGAGGCGATATCTGGCCGTCGATGCCCTGAACATGAGCAAGAAGGCAGGCCGTGGGGTGCGGGGTCGCAGTTAGGCTGGGAAGGCAGCACTTGAACTTGTCAATACCAGCCGCCCCCTTTTTTTCCAGACAAAGGGGTGTGAGCCCGCCTGGGGATGAGGCCTGGTGTGGTGGCAACTGACCTTAGCTTCGCGGGCTGACAGCTCTGGCCCAGGGCGGTATGTAATTCTGGCTCGACCTGGGACAGGGTCCAGCCAGGCCCAACCTGGCGGAGGCCAGGTCTGGGTGCGAGGGGGGTCTAGGGACTGCGTGAGGAGGTGAGGTGCCATGCTGAGCGGTGGCGCCTCGTGTGTGTCTTGACAGGCAGGGGCTGGAAGTGGACAAAATTGACCCACGCTTTATTTTCCAGGTGGCAGTGCTCCCTTTTGGACTTTTCCTGTAGGTTCCGTGCTAACCTCTTCTGTGAGCTCACTCTGCCCCTCCTCCTCCTCCTCCTCCCTTTAACTCCCTCGAGGTTCCCCATTGGCTTAAGCGTTGCTTCTGGTAATCTGGTAAGCCCGGGAAGTTGCTCCAGTCCCTGTTGGAGTCGCTACTGCTGGTAATCCTGAAGGAGGAGAGGCCTCCCCTGTTGAGACTCTGGGTGGGTGGGTGGCTTGCAGGTGGAGGGGGTGGGGCGTGGATGTGCACCCCCCGGGTGGGCCCGCGGCCATGGTACCAGCTGAAAGCTACGCAAATGCCCTCATAGATGGTGGTTTGTTGCTGTAGTGTTCATCATGGCGAGCTGATGGCACCAGGAGGATGGAGCCTCTGGCCAGTGTGAGTCCTAGCAGTGCAGGAGGGGAGACCCTGGAGGAGAGAGCCCGCTAAATTGATGTCTGCAGATTGAATTTCCAGAGGCTTAGGAGGAGGAAGTTCTCCAATGTCCCGTGTCCAGGCCTTTGGCTCAGGAAGCCCTGTATTCAGGAGGCCACCCTTTAAGGGTGTCAATGAGCTTGTGAGAGGGTGATGGTTCCAAGTCCCAAGCAGATGCATCCGGGGTCGTCAGCAGATGTGTTGAGTGTTTGATCCGGAGCACCAACCTCTGGGCACGTTGAATGCCAGCAGGCACCTCTCCTGTTCGGGGGTGGAGGTTTTCCGATGGCTGCGTCTTCTAATGCCTCCTGTCATGCTAGTTCTGTGCTACGAACCTTGCTTCCAGAAGACTCTTAAGATGTAGAACATTTTAACGCTGATGCCTCAACGCAGAGTGATGCCTGGTAGATGGAGCTTGCAGATGGAGCCCAGGGACCCTGTCATCTACTCACCTGCCTGCCCCAGCCTCGGTCGGAGGAATTCTTAGTGTGATGATGTAACCTGTGTGTCCAGAAAGTGTTCACTGTTGAAACGCCTTAGGCCAATACTCACAGGAAGCCACCTGGCAAACTGAGGGGGTCATTGTGTTGTGCTGTGGTCTGGGAGGGTACAAGGTGGTGCTGCGCAAGTGGGGATAGAAATGCAGGTCTGGTTTGGGCATTCCTACTACAAGAAGGGTGTGGTCAGGGTACTGATAGTGGTCTCTGCTCCATCTTGCCCTATATACTTTTTAACTTGAATTTTATTTGATTAGATTTCAAAGTAATTTTAGTCTTCAAATGAGGGGACCCCGAGGTGAGGGATTGGGAGGGAGGGGATGATTTGGCCCTGGCGCTGGGGAGGGGGAGGGGGGGGAAGAAAGGGAGAGGGTTGTGATTAGGTGTGGGTTCTGTAGGGTGGGAGTGGTTGGGAGTGTGGGTCGGAGGGAATTTTCTGGAGAAAATATCAATAGGCAGGGCTCTGGGGAGTTACGTAGAGCTCGTGGGAGAAGAAAATGTTAGTTAATAGTAAATTGTTAACGCTAGTTGTTGCTTGGAAGCGCAAAAGTTGCAAGTTTTCTAAAATGCTTAGAACTCTTAATTGCAGGAAGGAAAGCAAAATTGTTTTGCTTTGCAAGATGGGGAGGACTGGAAAAGGTAACAGCCTGGTTATCAGTCACTTGAAAGACTGGTGTCAGGAACTTGCATCAGCAGATTAACATCTTTCAATGCTTTGCAAGCTTCAAAGTGGTTTATCTGAATGTGTGCAGAATCCCGAGCAGTCTTAAGACTCTAAATACCCAAATTGAGGGCTTTTGAAGTATTATGTTTCAGATTTTGGTGTGCACTTAAGATGGTCAGATGTACTTTTCTGGGTTTGTAACATCTTAGCTGAGGTTTTGCAATTGAAAACTTGAGCAGAGTGAAAATGGTTTCTAGAGGAATTCCGTATGTCATCTCCTGCCTTATTAAACACATCTCTTGTCTTTGTGATTGGTACTACTACCAGCAGAAGTTTTTCCCATTAACCTTTTTGTCCTTGTTGTGACAAGAAGAACGTAATAACCTTGTGACCGCTGTCGTCACTGTATGTTTGGTAGCAAATAGTCCATGTATCCTTGATAAGGTTTCAGTTCATAATCCGCTGTATCTTTAGTTTGAATGTTTGTGCTTTTTAAGTGACCCAGTACTTCATGTGGGAAACTATTAAACCAAAATCAGTTTTGGAAATGAGAACAAAACTAGGGCTGGGCAAGTGCTTTAGATACGGTCTGTAAAAATCCAGGGGAAGATCTGAACTCTTATGATACTTGTTTCTGAGATGCACCTATTTAATTGGTAAAGTAACAGCTGTGGCTTTTTATCATCTCCCTTTTCCCCTTAAATTTACTTTGAAAATTTTAAATCCCCACTACTTATTACCCTTGGGAGTTACGTCTTCTGGGTTCTTTGGTGATGTTTAAAAATATGTTGACATGTAGTTCAATTCTTAACCTGTGACTTGGGCATGATGCGGTGACTCATTGGAATTTGAAGAGCACCAGTCACGCGTGTCGGGAAATATGTTGCGTTATGCTGTTGTGAAATCGAAACTGTAAAAGTAGGTGGTGAAAATCACGTCTCTGTTGCTCTGTGTGGTGAGAACCAGTCCTGTTGCATCATGTATGATGACTAATCATTTTTCTTCCCCTTCACAGCACAAATAAAGGTTTGAGTTCTAAACTCAAAAAAAAAAAAAAAAAAAAAAAAAAAAAA
